# Application of Data Mining for the Prediction of Mortality and Occurrence of Complications for Gastric Cancer Patients

**DOI:** 10.3390/e21121163

**Published:** 2019-11-28

**Authors:** Cristiana Neto, Maria Brito, Vítor Lopes, Hugo Peixoto, António Abelha, José Machado

**Affiliations:** 1 Algoritmi Research Center, University of Minho, 4710-057 Braga, Portugal; cristiana.neto@algoritmi.uminho.pt (C.N.); a73580@alunos.uminho.pt (M.B.); h.peixoto@di.uminho.pt (H.P.); abelha@di.uminho.pt (A.A.); 2 São João Hospital Center, 4200-319 Porto, Portugal; vitor.nuno.lopes@chsj.min-saude.pt

**Keywords:** healthcare, gastric cancer, knowledge discovery in databases, data mining, classification, prediction, clinical decision support systems, CRISP-DM, WEKA

## Abstract

The development of malign cells that can grow in any part of the stomach, known as gastric cancer, is one of the most common causes of death worldwide. In order to increase the survival rate in patients with this condition, it is essential to improve the decision-making process leading to a better and more efficient selection of treatment strategies. Nowadays, with the large amount of information present in hospital institutions, it is possible to use data mining algorithms to improve the healthcare delivery. Thus, this study, using the CRISP methodology, aims to predict not only the mortality associated with this disease, but also the occurrence of any complication following surgery. A set of classification models were tested and compared in order to improve the prediction accuracy. The study showed that, on one hand, the J48 algorithm using oversampling is the best technique to predict the mortality in gastric cancer patients, with an accuracy of approximately 74%. On the other hand, the rain forest algorithm using oversampling presents the best results when predicting the possible occurrence of complications among gastric cancer patients after their in-hospital stays, with an accuracy of approximately 83%.

## 1. Introduction

Many aspects that were previously unknown to healthcare professionals are now being revealed by the data generated by healthcare, improving the quality of medical procedures or treatment strategies [1]. Healthcare facilities like hospitals produce large amounts of heterogeneous data every day, since it includes diverse sources, data types and formats. This heterogeneity of healthcare data leads to the need of a rigorous observation of this data in order to assess its quality and identify possible problems that need to be solved. Since the data are so complex, it is practically impossible to analyze it with traditional tools and methods [2]. This complexity calls for more sophisticated techniques that are able to manage and produce meaningful knowledge. That way, the healthcare services records can serve as a way of assessing their quality and the patient’s satisfaction [3]. Thus, the use of data technologies like data mining (DM) has become essential in healthcare.

DM is a process that refers to the extraction of useful information from vast amounts of data [4]. It is used to find hidden patterns and uncover unknown correlations that are not obvious when observing the data with the naked eye [5]. Thus, DM can greatly benefit the healthcare industry by creating an environment rich in meaningful knowledge. Hence, using DM to support healthcare professionals in decision-making is crucial to ensure a good healthcare delivery [4].

One of the most common causes of death worldwide is gastric cancer. It is the fourth most frequently occurring cancer in men and the seventh most commonly occurring cancer in women. According to the World Cancer Research Fund (WCRF) [6] there were over 1 million new cases and an estimated 783,000 deaths related to gastric cancer in 2018. The greatest incidence rates are recorded in Eastern Asia (countries like South Korea, Mongolia and Japan occupy the first three spots), whereas in Northern America and Europe the rates are generally low [7].

Despite big advances in technologies and healthcare that provide better and more accurate diagnoses, this cancer, while registering a decreasing trend worldwide, remains among the most deadly malignancies [8]. There are a lot of factors that may influence the occurrence of this type of cancer. It is strongly suggested that general bad eating and drinking habits contribute to it. The consumption of alcoholic drinks and a diet rich in salty foods are among the most dangerous causes for gastric cancer. Smoking also plays a part in raising the risk of its occurrence [9].

Since this disease’s symptoms are not specific, they are often overlooked and may have other reasons besides gastric cancer. Initial signs may be indigestion, bloated feeling, slight nausea, and loss of appetite. A diagnosis at this early stage enhance the treatment opportunities to fight the cancer and can save a lot of lives. With the evolution of the condition and tumor growth, other symptoms, often more serious, start to manifest, such as stomach pain, vomiting, weight loss and constipation. Since the symptoms are considered ambiguous and it is not typical to do routine screenings, this cancer is often detected at later stages, strengthening the high mortality rates all over the world [8].

The focus of this study is the prediction of the mortality of patients that suffer from this gastric malignancy as well as the occurrence of complications after the patients’ hospital stays using DM techniques. The goal is to analyze the data available and the results obtained and make comparisons among different classifiers as to draw conclusions about them.

This paper is divided in seven sections. The current introduction section is proceeded by the state-of-the-art and by some works related to gastric cancer are mentioned. The fourth section is the methodology and methods, followed by a section that includes the phases of the data mining process. In the sixth section, the results obtained are compared and discussed. Finally, in the last section, some conclusions are drawn and the future work that will entail this DM project is revealed.

## 2. State of Art

In order to provide a deeper understanding of the context and importance of this study, this section provides the general background related to the associated research field. Thus, some concepts like knowledge discovery in databases (KDD), machine learning (ML) and DM are dissected and their association with the healthcare field leads to the introduction of the clinical decision support systems (CDSS).

### 2.1. Knowledge Discovery in Databases

Over the years, the rapid growth of digitization and computerization of processes in health institutions, as well as the large number of transactions that are performed daily in these environments, led to the production and collection of large amounts of data. This exponential increase in the amount of data stored by hospital institutions has raised the need to transform this data into relevant and useful information for the institution, leading to more efficient decision-making processes. This urgent need of extracting knowledge from the growing amount of digital data propelled the use of new computational theories and tools. This area is known as KDD [4,10,11].

**Figure 1:**
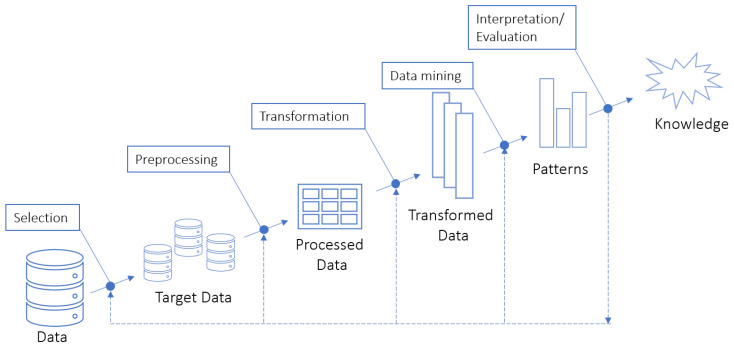
Phases of the KDD process.

According to Fayyad et al. [12], the KDD process consists of several phases and begins with the analysis of the application domain and the objectives to be accomplished, and this process is divided into 5 phases, represented on Figure 1.

The first step of the process is to choose the base to be mined, which can be data samples, subsets of variables up to large masses of data. The preprocessing phase aims to eliminate noise, missing values and illegitimate values. The data transformation step depends on the search objective and the algorithm to be applied, because it defines the limitations to be imposed on the database [11,12]. Improving data quality is important for better results, thus ensuring better quality in discovered patterns.

After completing the previous phases, DM is applied. This is the most important phase of the KDD process.

### 2.2. Data Mining

DM is the process of using machine learning techniques and statistical and mathematical functions to automatically extract potentially useful information from data in a way that is understandable to users. It can reveal the patterns and relationships among large amounts of data in a single or several data sets. The knowledge achieved can adopt various forms of representation, such as equations, trees or graphs, patterns or correlations [13].

DM methods can be divided into two categories: supervised and unsupervised. The supervised methods are used to predict a value and require the specification of a target attribute, on the contrary unsupervised methods are applied to discover the intrinsic structure, patterns, or affinities between the data [14].

The definition of the mining technique to be applied is closely related to the mining task to be performed, as this task defines the relationship between the data, ie the model. DM tasks are the types of discovery to perform in a database, that is, the information to extract. To determine which task to solve, it is important to have a good knowledge of the application domain and to know the type of information to obtain.

Therefore, DM includes two main types of techniques: descriptive and predictive. An example of descriptive techniques are the clustering techniques that are responsible for discovering information hidden in data. On the other hand, examples of predictive techniques are classification and regression techniques, that are used to retrieve new information from existing data [15,16,17]. The focal point of this paper are predictive techniques, more specifically, classification techniques.

Thus, there are many applications for DM, since it is greatly adaptable to distinct businesses and goals. They can go from retail stores, hospitals and banks to insurance or airline companies. The acquired knowledge during the DM process can also be used to support the decision-making process in various processes, e.g., in medicine—in the diagnosis phase, a correct and rapid analysis of this large volume of data is important for the identification of pathologies.

### 2.3. Clinical Decision Support Systems

To accomplish these goals, CDSS use clinical knowledge that is incorporated into the system helping professionals to analyze patient data, as well as decision-making. This knowledge used to maintain these systems is often extracted through DM techniques that, as mentioned before, are used to analyze and explore data with the aim of discovering patterns that might be helpful for decision-making [18].

## 3. Related Work

The improvement of gastric cancer diagnosis, mortality and complications rates have always been one of the most common work themes when it comes to the application of DM techniques in healthcare. Thus, some of the existing works have been studied prior to the conception of this paper.

Lee [15] applied DM techniques in order to create a prediction process for the occurrence of postoperative complications on gastric cancer patients. They have developed artificial neural networks (ANN) and compared their results with those of the traditional logistic regression (LR) approach, where they’ve achieved an average correct classification rate of 84.16% with ANN in contrast with 82.4% of LR.

Polaka et al. [16] planned various approaches for diagnosing gastric cancer using the original dataset and datasets with subsets of features. The best results were obtained for the dataset using attribute subsets selected with the wrapper approach. Four different models were tested, where C4.5 obtained 74.7% of accuracy, as well as CART. The RIPPER algorithm produced an accuracy of 73.9%, while the multilayer perceptron got the best results with 79.6%.

Hosein Zadeh et al. [17] used an optimized multivariate imputation by chained equations (MICE) technique to predict the chances of survival in gastric cancer patients. Three different techniques were executed: the first one, which consisted in the application of logistic regression, obtained 63.03% of accuracy, while the second technique that used a not optimized MICE algorithm earned an accuracy value of 66.14%. Finally, the third approach with the optimized MICE algorithm produced results with 72.57% of accuracy.

Mohammadzadeh et al. [19] carried out a study aimed to develop a decision model for predicting the probability of mortality in gastric cancer patients also identifying the most important factors influencing the mortality of patients who suffer from this disease. Regarding the effective factors on mortality of gastric cancer, the determined factors were diabetes, ethnicity, tobacco, tumor size, surgery, pathological stage, age at diagnosis, exposure to chemical weapons and alcohol consumption. The accuracy of developed decision tree was 74%.

## 4. Methodology and Methods

### 4.1. Methodology

The reference model used during the development of this study was cross-industry standard process for DM, most commonly known as CRISP-DM.

**Figure 2:**
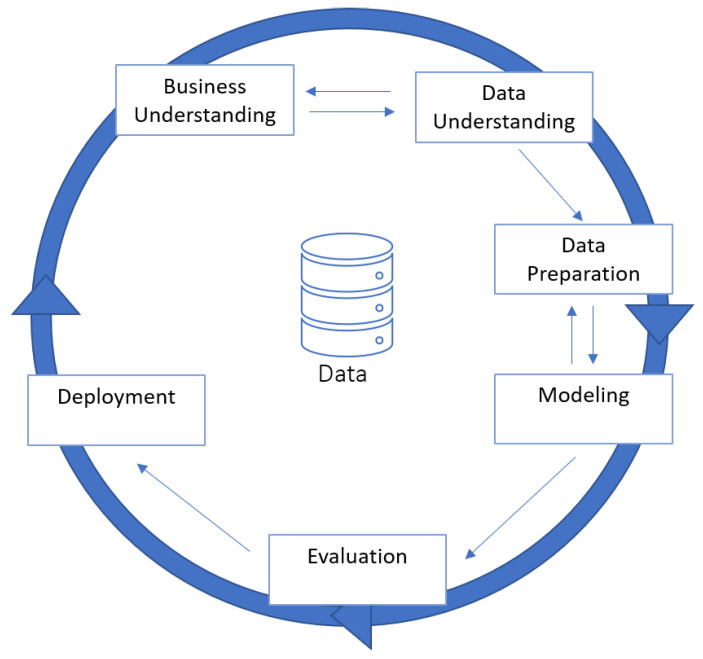
Phases of the CRISP-DM model.

The CRISP-DM methodology provides a structured approach to planning a DM project and is a six phase hierarchical process, divided in the following steps: business understanding, data understanding, data preparation, modelling, and evaluation and deployment, as shown on Figure 2 [20,21]. The Section 5 describes in detail the application of all this steps in the context of this study.

### 4.2. Methods

In order to analyze and explore the available data and to induce the data mining models (DMM), the chosen ML software was Waikato Environment for Knowledge Analysis (WEKA). During the execution of this study, five modelling techniques were used with WEKA in order to induce the DM models, namely: random forest (RF), J48, simple logistic (SL), Bayes net (BN) and PART. This study includes ensemble techniques Bagging and adaptive boosting (Adaboost) using some of the mentioned algorithms. It is important to note that in this study the application of oversampling using synthetic minority oversampling (SMOTE) was also tested.

#### 4.2.1. Random Forest

RF is an ensemble learning method for classification which operates by constructing a multitude of decision trees. Initially, a bootstrap sample from the training data was selected (random sample obtained with replacement) with the goal of inducing a decision tree (DT). The repetition of this step was performed until an ensemble of DTs was created, each one of them having its own prediction value. Thus, the final prediction was achieved by combining the output from all trees, which corresponds to the most frequent output obtained by the ensemble. RF could correct for decision trees’ habit of overfitting to their training set making it a very efficient and accurate classifier [22,23].

#### 4.2.2. J48

The J48 algorithm used greedy technology to induce DTs for further classification. J48 generated decision trees, where each tree node evaluated the existence or significance of each individual attribute. Decision trees were built from top to bottom by choosing the most appropriate attribute for each situation. Once the attribute was chosen, the training data was divided into subgroups, corresponding to the different attribute values and the process was repeated for each subgroup until a large part of the attributes in each subgroup belonged to a single class. It is important to note that the J48 classifier implemented by WEKA corresponds to an open source Java implementation of the C4.5 algorithm and is considered one of the most powerful and commonly used DT classifier [24,25,26].

#### 4.2.3. Simple Logistic

SL is a classifier for building linear logistic regression models. LogitBoost with simple regression functions as base learners is used for fitting the logistic models. The optimal number of LogitBoost iterations to perform is cross-validated, which leads to automatic attribute selection [27,28].

#### 4.2.4. Bayes Net

BN is a base class for a Bayes network classifier. A Bayesian is a graphical model for probabilistic relationships among a set of variables and is composed of directed acyclic graphs. It also provides data structures like conditional probability distributions, network structure, etc and facilities common to Bayes network learning algorithms like K2 and B [29,30].

#### 4.2.5. PART

PART is a partial decision tree algorithm, which is a combination version of C4.5 and RIPPER algorithms, developed to try to avoid their respective problems. The main specialty of the PART algorithm is that it does not need to perform global optimization like C4.5 and RIPPER to produce the appropriate rules. The fact that PART adopts the separate-and-conquer strategy, building a rule, removing the instances it covers and continuing creating rules recursively for the remaining instances until none are left, is a big advantage [31,32].

#### 4.2.6. Bagging

Bagging is one of the popular ensemble methods proposed by Freund and Schapire [33] for improving classifiers. Bagging is based on bootstrapping and aggregating concepts, integrating the benefits of both approaches [34]. In Bagging, the training set is sampled generating random independent bootstrap replicates. In addition, the classifier on each of these is constructed and aggregated by a simple majority vote in the final decision rule [35].

#### 4.2.7. AdaBoost

Freund and Schapire [33] also proposed Adaboost, a shortening of adaptive boosting. This algorithm stands out mainly due to its potential, flexibility, and simplicity to be implemented in different scenarios. It is an iterative process that produces a strong classifier which consists of a sequence of weighted classifiers that complement one another. AdaBoost achieves its ultimate classifier goal by sequentially introducing new models in order to compensate for the misclassified instances in previous iterations [35].

#### 4.2.8. Sythetic Minority Oversampling (SMOTE)

SMOTE is a popular oversampling technique (which replicates examples from the minority class). It creates new samples based on the interpolation of minority class instances. Based on k nearest neighbours (kNN), it randomly selects samples from minority classes and generates the new ones [36].

## 5. Data Mining Process

### 5.1. Business Understanding

Cancer affects millions of people all over the world and is one of the biggest threats to people’s lives and life quality. Gastric cancer is one of the most common causes of cancer related deaths, behind, for example, lung cancer [37]. The prognostic is usually not favorable to the survival of patients, since there is only a probability of less than 30% survival upon diagnosis in Europe [37]. However, in Japan this rate goes up to 90% thanks to early examinations and tumor resections [38].

This malignancy presents no specific symptoms in early stages, which causes delayed diagnoses that lead to the high mortality of patients. In advanced stages, the patient may feel a variety of more serious symptoms, like abdominal pain, indigestion, severe nausea and inexplicable weight loss [38]. By the time these symptoms appear, the cancer has already developed to more dangerous stages. When the tumor is diagnosed, it is often too late for any curative medical procedure to take place. There are various objectives with this study, such as:

- Promote early examinations among the general population in order to avoid late gastric cancer diagnoses that often lead to the patient’s death
- Predict the probability of mortality after the surgery
- Predict the occurrence of complications after in-hospital stays for gastric cancer patients

Thus, this study aims to improve many aspects related to gastric cancer and the way it affects the patients’ lives. The focus falls on their hospital admissions and possible complications that may occur related or not to the tumor. The procedures performed and the patient’s health status after the hospital stay are also subjects of this work.

The first item is related to the healthcare business goals. The improvement of the quality of the medical services provided is one of the most crucial aspects in this industry. This translates into an increment on the survival rates of patients, in this case patients that suffer from gastric cancer.

The rest of the goals listed are related to the objectives inherent to the DM process. Through the application and refinement of DM techniques these objectives will provide a substantial help to healthcare professionals.

### 5.2. Data Understanding

The data used for this study was collected from a Portuguese hospital and is related to patients with gastric cancer. It includes over 60 variables with information about the patients’ admission, stay at the hospital, possible complications and the result of the performed procedure related to 154 patients.

### 5.3. Data Preparation

The original dataset provided had a lot of attributes with high percentages of missing values. When it comes to the numerical variables, half of the attributes have over 45% of missing or null values. This makes them not useful to study or to subject them to ML algorithms, since they offer little to no meaningful information. Consequently, these attributes were removed from the dataset. Moreover, after a careful analysis, it was detected that there were extremely similar attributes, even presenting the same values. As such, one of those attributes was also removed from the dataset, leaving only one of them in the dataset in order to avoid any redundancy. Also, some of the attributes refer to technical aspects related to the extraction of the data, so they were removed from the dataset as well. The categorical attributes were submitted to the same process.

After the data cleaning, three more features were created derived from existing attributes. These new features refer to the number of postoperative complications registered, to the occurrence of complications 30 days after the in-hospital stay and to the death of patients.

The final result was a dataset with 33 features (4 numeric and 29 categorical)(use Case one). However, in order to analyze alternative approaches with fewer attributes, three more datasets were created.

The first one (use Case two) was created with attribute selection performed by the OneR algorithm, where 19 attributes were selected (1 numeric and 18 categorical). Whereas, the second dataset (Use Case 3) included a subset of features that were selected using the Relief algorithm. This one was composed of 20 attributes, from which one was numeric and the rest categorical. On the other hand, the features selected for the third dataset (Use Case 4) were chosen based on the Pearson’s correlation method. This subset of features was comprised of 21 attributes, where 2 of them were numeric and 19 were categorical. The summary of the characteristics of the datasets can be checked on the Table 1.

### 5.4. Modeling

The first proposed goal was to predict the mortality of gastric cancer patients that were admitted to the hospital. Based on the health status available, as well as info about the performed surgery and its outcome, the models will predict if it’s more likely that the patient will survive or pass away. In this case, four datasets (the original - after the data preparation - and three more that resulted of feature selection) were tested. Thus, the classification process included four scenarios that contemplated distinct set of features.

On the other hand, the second goal was to predict the occurrence of complications after hospital stays. In this case, features related to the patients’ morbidity and survival, and complications’ rank were removed, along with information about the possible existence of complications. These attributes were eliminated in order to ensure an unbiased and correct prediction.

In order to assure that the models are assuming most of the patterns from the data correctly, and are low on bias and variance, the usage of cross validation came into action. Cross validation provided ample data to train the model and also leaving a lot of data to test it. For this study 10-folder cross validation was used, dividing the dataset into 10 folds, and the holdout method was repeated 10 times, such that each time, one of the 10 subsets was used as the test subset and the other nine subsets are put together to form the training set.

The classifiers selected for this study were RF, J48, Simple Logistic, BN, and PART. In addition, the algorithm AdaBoost and Bagging were also executed in conjunction with the first three models already mentioned. In this DM approach, specifically when using decision trees, the main criterion for selecting a variable to make a decision was the dependence of a variable on the class variable. There was no differentiation between direct dependence and indirect dependence (intermediated by other variables). Such distinction did not make a difference for classification, because trees based on direct dependence or indirect dependence were very likely to result in the same classification. Nevertheless, if these approaches are used to intervene in a system of the real world, indirect dependence may not have impact, while direct dependence can.

Finally, two data approaches were tested: with and without oversampling (using SMOTE).

At this stage, the DMMs were constructed using the WEKA software. A DMM can be compose by a target variable (T), a scenario (S), a data mining technique (DMT), a data approach (DA) and a sampling method (SM). Regarding the DM mortality (DM1):

- T = {Mortality}
- S = {S1, S2, S3, S4}
- DMT = {RF, J48, J48 using Laplace correction, SL, BN, PART, AdaBoost + RF, AdaBoost + J48, AdaBoost + J48 using Laplace correction, AdaBoost + SL, Bagging + RF, Bagging + J48, Bagging + J48a using Laplace correction, Bagging + SL}
- DA = {With oversampling, without oversampling}
- SM = {Cross-validation 10 Folds}

- S1 = {all attributes (Use Case 1)}
- S2 = {19 attributes selected by the OneR algorithm (Use Case 2)}
- S3 = {20 attributes selected by the Relief algorithm (Use Case 3)}
- S4 = {21 attributes selected by the Pearson’s correlation method (Use Case 4)}

For DM surgery complications (DM2):

- T = {Surgery complications}
- S = {S1}
- DMT = {RF, J48, J48 using Laplace correction, SL, BN, PART, AdaBoost + RF, AdaBoost + J48, AdaBoost + J48 using Laplace correction, AdaBoost + SL, Bagging + RF, Bagging + J48, Bagging + J48a using Laplace correction, Bagging + SL}
- DA = {Without oversampling}
- SM = {Cross validation 10 Folds}
- S1 = {22 attributes (without bias attributes)}

Thus, the induced DMM were: 

DMM1 = one target, four scenarios, 14 DM techniques, one sampling dethod, two data approaches

DMM2 = one target, one scenario, 14 DM techniques, one sampling method, two data approaches 

In total, 140 simulations were performed (1 × 4 × 14 × 1 × 2 for DMM1 plus 1 × 1 × 14 × 1 × 2 for DM2).

### 5.5. Evaluation

Once the modeling phase was concluded, the chosen classifiers were put to test in order to evaluate and compare their results. The metrics used were accuracy, precision, F-measure and recall. They are defined as such:

(1) Accuracy=(TP+TN)=(TP+FP+TN+FN)

(2) Precision=TP=(TP+FP)

(3) FMeasure=2x((PR+RC)=(PRxRC))

(4) Recall=TP=(TP+FN)

where TP = true positives, TN = true negatives, FP = false positives, FN = false negatives PR = precision, and RC = recall.

The area under the ROC curve (AUC) metric was also used. ROC is a probability curve and AUC represents degree or measure of separability that represents how much the model is capable of distinguishing between classes.

In order to ease the understanding of the results obtained, they are divided by scenarios as shown below.

#### 5.5.1. Mortality Prediction Results

The Table 2 presents the results obtained during the classification process using the original dataset for mortality, after the data preparation. It is important to note that for each DM technique two data approaches were tested: with and without oversampling.

The Table 3 exposes the results obtained for the prediction of gastric cancer patients’ mortality using the feature selection technique that evaluates the worth of a feature using the OneR algorithm.

The Table 4 presents the results obtained for the prediction of gastric cancer patients’ mortality using the feature selection method that evaluates the worth of an attribute using the Relief algorithm.

In the Table 5, the results obtained for the prediction of gastric cancer patients’ mortality using the feature selection that evaluates the worth of attributes by using the Pearson’s correlation are presented.

#### 5.5.2. Prediction Results for the Occurrence of Complications

Table 6 shows the results of the prediction of the occurrence of complications after surgery.

## 6. Discussion

### 6.1. Predict the Mortality of Gastric Cancer Patients

**Figure 3:**
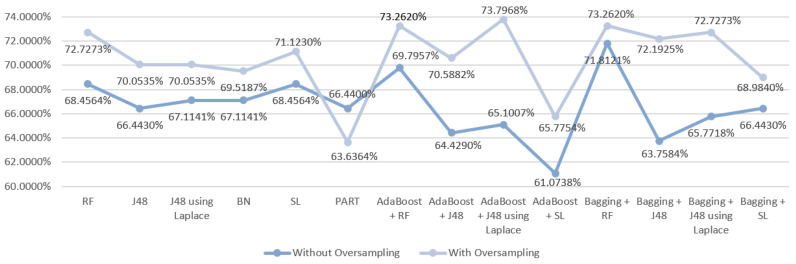
Comparative graph for the results obtained in the mortality prediction for S1.

Regarding the first scenario, a first analysis of the results (Figure 3) revealed that the use of oversampling improved them in 13/14 of the tested DM techniques, as it only worsened with the PART algorithm. Without the use of oversampling, SL produced the best result, which despite presenting the same accuracy as the RF model (68.4564%) showed better results for the other metrics in comparison with RF. The best result for this scenario was obtained using oversampling by the ensemble technique Boosting with J48 using Laplace correction, achieving an accuracy of 73.7968%.

**Figure 4:**
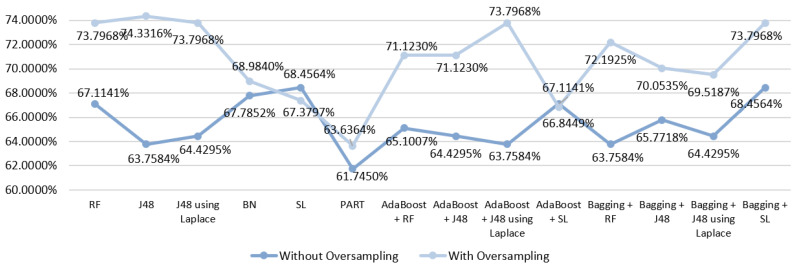
Comparative graph for the results obtained in the mortality prediction for S2.

Using a dataset with fewer features than the original one (scenario two), the initial best results were achieved with the SL algorithm that produced an accuracy of 68.4564%. However, as it can be seen in Figure 4, better values were obtained with the usage of oversampling, except when using the SL algoritm (alone and ensemble with Adaboost). So, the best result obtained was 74.3316% using the J48 algorithm with oversampling.

**Figure 5:**
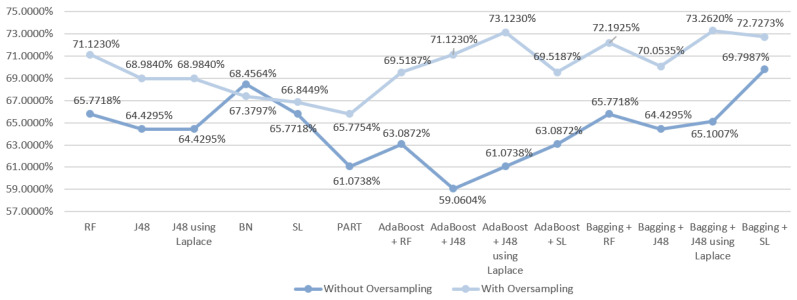
Comparative graph for the results obtained in the mortality prediction for S3.

In the third scenario, the results that were obtained from the execution of the selected models showed that initially the best result was obtained by the ensemble technique Boosting with SL resulting in an accuracy of 69.7987%. Observing the Figure 5, it is notable that the use of oversampling improved the results obtained in 13/14 of the tested DM techniques, just like in S1, but this time the exception was the SL algorithm. Thus, the best accuracy value for this scenario was 73.2620%, obtained using the ensemble technique Bagging with J48 using Laplace correction.

**Figure 6:**
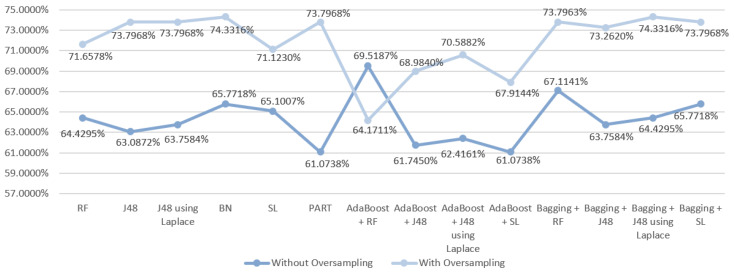
Comparative graph for the results obtained in the mortality prediction for S4.

Finally, in the last scenario of mortality prediction, the Figure 6 shows that, like S1, S2 and S3, the use of oversampling also increased the accuracy values in 13/14 of the applied algorithms. This time the exception was the ensemble technique Boosting with RF that presented the highest accuracy value (69.5187%) before using oversampling but decreasing after.

With the usage of oversampling, the results that were obtained from the execution of the selected models showed that both PART and the ensemble technique Bagging with J48 using Laplace correction produced the best accuracy of 74.3316%.

When compared to the results obtained with the datasets that were submitted to feature selection methods, it’s possible to conclude that the original dataset (S1) produced better overall results for the selected metrics without the use of oversampling, as can be seen on the Table 7, Table 8 and Table 9. However, that observation changed with the application of oversampling since the results increased in other scenarios.

Overall, the best result was obtained with the second scenario, ie using the attributes selected by the OneR algorithm, which achieved an accuracy of 74.3316%, an accuracy of 0.744, an F-measure of 0.743, a recall of 0.743 and an AUC of 0.826 using the J48 algorithm and the oversampling data technique.

Moreover, the results obtained for AUC highlight two algorithms: BN and RF (although combined with other techniques), 0.879 being the best AUC result, obtained by Bagging with RF. However, most of the algorithms obtained an AUC close to 1. A good model has AUC near to 1 which means it has a good measure of separability.

The results obtained were not very high, due to the multitude of reasons that may lead to a patient’s death. These include factors not directly linked to the gastric cancer or deaths that took place because the patient was already palliative.

### 6.2. Predict the Occurrence of Complications after In-Hospital Stays for Gastric Cancer Patients

**Figure 7:**
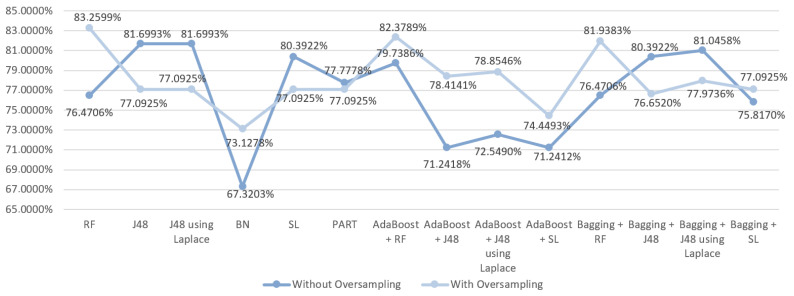
Comparative graph for the results obtained in the complications prediction.

When it comes to the prediction of complications after a hospital stay for gastric cancer, the results obtained were more satisfactory, as can be observed on Figure 7. The reason for that is that it is considerably easier to anticipate if a patient will suffer from any complications or disabilities following a surgery by observing the health status available. As such, initially, the best accuracy value was recorded for the J48 algorithm (81.6993%). In contrast to mortality prediction, in this case the use of oversampling only improved in 8/14 algorithms. Nevertheless, the best final outcome for predicting complications was obtained using oversampling, achieving an accuracy of 83.2599 % with the RF algorithm, that also presented the best AUC value of 0.909.

### 6.3. Summary

The best results achieved with this study, previously described, are summarised in Table 10, where the two defined objectives are represented (DMM1 and DMM2). The obtained results, especially for the mortality prediction, are in accordance with the reviewed literature.

Relating the two main objectives of this project, although pertinent, the attribute related to the occurrence of complications after surgery was not considered crucial in predicting mortality. Although the occurrence of complications after surgery may be directly related to mortality, in the case of this dataset most patients survived even after the occurrence of complications.

The use of data mining in the healthcare setting can lead to several outcomes, not strictly related to the classification problems presented hereby. With this kind of approach, new scientific knowledge can also be achieved, when understanding the contribution of the selected predictors for the response variable. The several variables identified as predictive in terms of prognosis have a well-established relationship in patients operated due to gastric cancer among medical literature.

TNM classification of malignant tumors and stage, for example, is usedby theoretical models and physicians in practical evaluation and as expected are part of the top attributes when predicting mortality and complications using data mining. The American Joint Committee on Cancer (AJCC)/TNM classification is widely used among different cancers as a staging score, with implications in terms of prognosis [39], as this study succeeds in confirming.

Other relevant attributes found by this analysis include the reason to search for medical care, which can vary since the presentation of the disease can go from weight loss, nausea, and other mild symptoms to anemia stigma, vomiting, and hematemesis (upper tract hemorrhage). One should take into account that the incidence of symptoms are usually suggestive of a more advanced stage, with a subsequent worse prognosis [40]. Last but not least, we also found that the post-operative recovery is an important predictor of the mortality of this cancer, since patients which demand ICU care, tend to have worse prognosis due to being hemodynamically unstable or having several or serious comorbidities [41].

## 7. Conclusions and Future Work

In the last years, KDD and, more specifically, DM techniques are becoming increasingly useful for processing and exploiting medical data. The useful information discovered and the patterns obtained with the application of these methods, analysing in real-time complex and heterogeneous data and make conclusions about it, can be used by health professionals to determine diagnoses, prognoses and treatments for patients in healthcare organizations.

What was an impossible task to execute in the past, it is now possible to submit millions and millions of medical records to an algorithm and receive relevant results. There are numerous software available to the general public that offers tools for data processing, reading it, cleaning it, preparing it for the application of algorithms, and even allowing to execute and refine the models.

This paper aimed to predict the mortality of gastric cancer patients based on their health status, data about the tumor and surgery information, as well as to make predictions about the possibility of occurrence of complications following a in-hospital stay using DM techniques. Considering the various reasons that may lead to the patient’s death, it becomes challenging to predict if the patient might perish or survive. There are a lot of aspects that influence this outcome that show no direct link to the cancer in question. A lot of patients, due to late diagnosis, face little to no chances of survival since no curative treatment can treat the tumor. These facts contribute to obtain the best accuracy values around 74%.

On the other hand, it is simpler to predict if a patient will suffer from complications after their hospital stay, since it is possible to rely more on the data available. Observing the data about the tumor (its localization, stage, size, lymph nodes and metastasis) and analysing the health status of the patient (given by the ASA score) among other factors, the prediction of the occurrence of complications becomes a more straightforward process. Hence, the accuracy obtained for this goal was around 83%.

Future work will consist in obtaining a larger dataset with more relevant data in order to improve the prediction process for both patients’ mortality and occurrence of complications. Others models will also be tested and their results compared with the ones already obtained. Also, an interesting future research would be to attempt to determine whether there exists a causal relationship between the different variables in the used dataset, and the usage of other state of the art machine learning algorithms such as deep neural networks. Finally, it would be interesting to use this work in a CDSS in order to assist healthcare professionals and, consequently, improve the healthcare delivery for patients with gastric cancer.

## Figures and Tables

**Table 1:** Summary of the datasets created for the prediction of mortality.

Use Case	# Attr	# Numeric Attr	# Categorical Attr
1	33	4	29
2	19	1	18
3	20	1	19
4	21	2	19

**Table 2:** Prediction results for the mortality for the first scenario.

DM Technique	Scenario	Data Approach	Accuracy (%)	Precision	F-Measure	Recall	AUC
RF	S1	Without oversampling	68.4564	0.670	0.674	0.685	0.815
		With oversampling	72.7273	0.723	0.717	0.727	0.859
J48	S1	Without oversampling	66.443	0.640	0.647	0.664	0.770
		With oversampling	70.0535	0.699	0.694	0.701	0.772
J48 ${}^{a}$	S1	Without oversampling	67.1141	0.645	0.652	0.671	0.755
		With oversampling	70.0535	0.699	0.694	0.701	0.801
BN	S1	Without oversampling	67.1141	0.676	0.672	0.761	0.808
		With oversampling	69.5187	0.693	0.694	0.695	0.837
SL	S1	Without oversampling	68.4564	0.683	0.681	0.685	0.792
		With oversampling	71.1230	0.706	0.707	0.711	0.821
PART	S1	Without oversampling	66.443	0.662	0.658	0.664	0.777
		With oversampling	63.6364	0.629	0.631	0.636	0.748
AdaBoost + RF	S1	Without oversampling	69.7957	0.686	0.688	0.698	0.821
		With oversampling	73.2620	0.726	0.725	0.733	0.862
AdaBoost + J48	S1	Without oversampling	64.4295	0.640	0.642	0.644	0.790
		With oversampling	70.5882	0.701	0.701	0.706	0.848
AdaBoost + J48 ${}^{a}$	S1	Without oversampling	65.1007	0.659	0.655	0.651	0.794
		With oversampling	73.7968	0.738	0.731	0.738	0.850
AdaBoost + SL	S1	Without oversampling	61.0738	0.619	0.614	0.611	0.693
		With oversampling	65.7754	0.662	0.659	0.658	0.783
Bagging + RF	S1	Without oversampling	71.8121	0.702	0.705	0.718	0.810
		With oversampling	73.2620	0.724	0.718	0.733	0.859
Bagging + J48	S1	Without oversampling	63.7584	0.605	0.616	0.638	0.800
		With oversampling	72.1925	0.716	0.718	0.722	0.840
Bagging + J48 ${}^{a}$	S1	Without oversampling	65.7718	0.637	0.643	0.658	0.801
		With oversampling	72.7273	0.722	0.723	0.727	0.840
Bagging + SL	S1	Without oversampling	66.443	0.656	0.660	0.664	0.780
		With oversampling	68.9840	0.690	0.689	0.690	0.834

${}^{a}$ Using Laplace correction.

**Table 3:** Prediction results for the mortality for the second scenario.

DM Technique	Scenario	Data Approach	Accuracy (%)	Precision	F-Measure	Recall	AUC
RF	S2	Without oversampling	67.1141	0.656	0.659	0.671	0.811
		With oversampling	73.7968	0.735	0.736	0.738	0.873
J48	S2	Without oversampling	63.7584	0.625	0.629	0.638	0.789
		With oversampling	74.3316	0.744	0.743	0.743	0.826
J48 ${}^{a}$	S2	Without oversampling	64.4295	0.629	0.634	0.644	0.761
		With oversampling	73.7968	0.738	0.738	0.738	0.821
BN	S2	Without oversampling	67.7852	0.675	0.676	0.678	0.820
		With oversampling	68.9840	0.687	0.687	0.690	0.836
SL	S2	Without oversampling	68.4564	0.682	0.682	0.685	0.793
		With oversampling	67.3797	0.667	0.669	0.674	0.804
PART	S2	Without oversampling	61.7450	0.603	0.604	0.617	0.754
		With oversampling	63.6364	0.632	0.633	0.636	0.776
AdaBoost + RF	S2	Without oversampling	65.1007	0.643	0.646	0.651	0.760
		With oversampling	71.1230	0.710	0.711	0.711	0.829
AdaBoost + J48	S2	Without oversampling	64.4295	0.643	0.643	0.644	0.779
		With oversampling	71.1230	0.709	0.710	0.711	0.868
AdaBoost + J48 ${}^{a}$	S2	Without oversampling	63.7584	0.634	0.636	0.638	0.778
		With oversampling	73.7968	0.733	0.734	0.738	0.878
AdaBoost + SL	S2	Without oversampling	67.1141	0.670	0.671	0.671	0.791
		With oversampling	66.8449	0.666	0.667	0.668	0.802
Bagging + RF	S2	Without oversampling	63.7584	0.616	0.623	0.638	0.804
		With oversampling	72.1925	0.718	0.719	0.722	0.879
Bagging + J48	S2	Without oversampling	65.7718	0.629	0.639	0.658	0.801
		With oversampling	70.0535	0.697	0.697	0.701	0.842
Bagging + J48 ${}^{a}$	S2	Without oversampling	64.4295	0.617	0.628	0.644	0.798
		With oversampling	69.5187	0.690	0.691	0.695	0.835
Bagging + SL	S2	Without oversampling	68.4564	0.672	0.676	0.685	0.806
		With oversampling	73.7968	0.734	0.733	0.738	0.848

${}^{a}$ Using Laplace correction.

**Table 4:** Prediction results for the class mortality for the third scenario.

DM Technique	Scenario	Data Approach	Accuracy (%)	Precision	F-Measure	Recall	AUC
RF	S3	Without oversampling	65.7718	0.644	0.646	0.658	0.805
		With oversampling	71.1230	0.699	0.700	0.711	0.863
J48	S3	Without oversampling	64.4295	0.625	0.632	0.644	0.781
		With oversampling	68.9840	0.682	0.683	0.690	0.795
J48 ${}^{a}$	S3	Without oversampling	64.4295	0.625	0.632	0.644	0.754
		With oversampling	68.9840	0.682	0.683	0.690	0.793
BN	S3	Without oversampling	68.4564	0.682	0.683	0.685	0.820
		With oversampling	67.3797	0.682	0.674	0.674	0.836
SL	S3	Without oversampling	65.7718	0.651	0.652	0.658	0.790
		With oversampling	66.8449	0.663	0.664	0.668	0.825
PART	S3	Without oversampling	61.0738	0.600	0.604	0.611	0.756
		With oversampling	65.7754	0.648	0.650	0.658	0.770
AdaBoost + RF	S3	Without oversampling	63.0872	0.614	0.621	0.631	0.789
		With oversampling	69.5187	0.688	0.691	0.695	0.830
AdaBoost + J48	S3	Without oversampling	59.0604	0.604	0.597	0.591	0.778
		With oversampling	71.1230	0.711	0.711	0.711	0.861
AdaBoost + J48 ${}^{a}$	S3	Without oversampling	61.0738	0.619	0.614	0.611	0.780
		With oversampling	73.1230	0.708	0.709	0.711	0.859
AdaBoost + SL	S3	Without oversampling	63.0872	0.617	0.623	0.631	0.766
		With oversampling	69.5187	0.692	0.693	0.665	0.815
Bagging + RF	S3	Without oversampling	65.7718	0.640	0.658	0.644	0.805
		With oversampling	72.1925	0.718	0.719	0.722	0.862
Bagging + J48	S3	Without oversampling	64.4295	0.611	0.624	0.644	0.800
		With oversampling	70.0535	0.697	0.697	0.701	0.845
Bagging + J48 ${}^{a}$	S3	Without oversampling	65.1007	0.623	0.633	0.651	0.801
		With oversampling	73.2620	0.728	0.729	0.733	0.841
Bagging + SL	S3	Without oversampling	69.7987	0.683	0.688	0.698	0.799
		With oversampling	72.7273	0.727	0.726	0.727	0.857

${}^{a}$ Using Laplace correction.

**Table 5:** Prediction results for the class mortality for the fourth scenario.

DM Technique	Scenario	Data Approach	Accuracy (%)	Precision	F-Measure	Recall	AUC
RF	S3	Without oversampling	64.4295	0.632	0.636	0.644	0.801
		With oversampling	71.6578	0.712	0.714	0.717	0.859
J48	S3	Without oversampling	63.0872	0.602	0.614	0.631	0.753
		With oversampling	73.7968	0.735	0.735	0.738	0.792
J48 ${}^{a}$	S3	Without oversampling	63.7584	0.606	0.619	0.638	0.734
		With oversampling	73.7968	0.735	0.735	0.738	0.797
BN	S3	Without oversampling	65.7718	0.653	0.655	0.658	0.812
		With oversampling	74.3316	0.740	0.741	0.743	0.842
SL	S3	Without oversampling	65.1007	0.653	0.655	0.658	0.795
		With oversampling	71.1230	0.709	0.708	0.711	0.809
PART	S3	Without oversampling	61.0738	0.603	0.606	0.611	0.735
		With oversampling	73.7968	0.735	0.735	0.738	0.782
AdaBoost + RF	S3	Without oversampling	69.5187	0.688	0.691	0.695	0.740
		With oversampling	64.1711	0.640	0.641	0.642	0.807
AdaBoost + J48	S3	Without oversampling	61.7450	0.609	0.613	0.617	0.790
		With oversampling	68.9840	0.687	0.688	0.690	0.854
AdaBoost + J48 ${}^{a}$	S3	Without oversampling	62.4161	0.622	0.621	0.624	0.787
		With oversampling	70.5882	0.705	0.706	0.706	0.859
AdaBoost + SL	S3	Without oversampling	61.0738	0.622	0.616	0.611	0.776
		With oversampling	67.9144	0.684	0.681	0.679	0.802
Bagging + RF	S3	Without oversampling	67.1141	0.653	0.658	0.671	0.803
		With oversampling	73.7963	0.732	0.734	0.738	0.864
Bagging + J48	S3	Without oversampling	63.7584	0.617	0.624	0.638	0.794
		With oversampling	73.2620	0.732	0.732	0.733	0.836
Bagging + J48 ${}^{a}$	S3	Without oversampling	64.4295	0.608	0.621	0.644	0.795
		With oversampling	74.3316	0.742	0.742	0.743	0.832
Bagging + SL	S3	Without oversampling	65.7718	0.648	0.652	0.658	0.800
		With oversampling	73.7968	0.733	0.735	0.738	0.856

${}^{a}$ Using Laplace correction.

**Table 6:** Prediction results for the occurrence of complications.

DM Technique	Scenario	Data Approach	Accuracy (%)	Precision	F-Measure	Recall	AUC
RF	S3	Without oversampling	76.4706	0.730	0.730	0.765	0.654
		With oversampling	83.2599	0.833	0.833	0.833	0.909
J48	S3	Without oversampling	81.6993	0.834	0.777	0.817	0.580
		With oversampling	77.0925	0.773	0.770	0.771	0.806
J48 ${}^{a}$	S3	Without oversampling	81.6993	0.834	0.777	0.817	0.580
		With oversampling	77.0925	0.773	0.770	0.771	0.825
BN	S3	Without oversampling	67.3203	0.708	0.687	0.673	0.690
		With oversampling	73.1278	0.733	0.731	0.731	0.822
SL	S3	Without oversampling	80.3922	0.807	0.761	0.804	0.662
		With oversampling	77.0925	0.771	0.771	0.771	0.826
PART	S3	Without oversampling	77.7778	0.753	0.754	0.778	0.655
		With oversampling	77.0925	0.775	0.770	0.771	0.794
AdaBoost + RF	S3	Without oversampling	79.7386	0.798	0.750	0.797	0.662
		With oversampling	82.3789	0.824	0.824	0.824	0.914
AdaBoost + J48	S3	Without oversampling	71.2418	0.696	0.703	0.712	0.640
		With oversampling	78.4141	0.785	0.784	0.784	0.868
AdaBoost + J48 ${}^{a}$	S3	Without oversampling	72.5490	0.705	0.713	0.725	0.635
		With oversampling	78.8546	0.794	0.788	0.789	0.868
AdaBoost + SL	S3	Without oversampling	71.2412	0.702	0.707	0.712	0.599
		With oversampling	74.4493	0.745	0.745	0.744	0.770
Bagging + RF	S3	Without oversampling	76.4706	0.725	0.698	0.765	0.681
		With oversampling	81.9383	0.819	0.819	0.819	0.908
Bagging + J48	S3	Without oversampling	80.3922	0.793	0.771	0.804	0.643
		With oversampling	76.6520	0.767	0.767	0.767	0.872
Bagging + J48 ${}^{a}$	S3	Without oversampling	81.0458	0.806	0.776	0.810	0.660
		With oversampling	77.9736	0.781	0.780	0.780	0.879
Bagging + SL	S3	Without oversampling	75.8170	0.736	0.743	0.758	0.641
		With oversampling	77.0925	0.771	0.771	0.771	0.852

${}^{a}$ Using Laplace correction.

**Table 7:** Summary of the best results for the prediction of mortality (Accuracy).

Scenario	Data Technique	Classifier	Accuracy (%)
S1	Without oversampling	Bagging with RF	71.8121
	With oversampling	Boosting with J48 using Laplace	73.7968
S2	Without oversampling	SL and Bagging with SL	68.4564
	With oversampling	J48	74.3316
S3	Without oversampling	Boosting with SL	69.7987
	With oversampling	Boosting with J48 using Laplace	73.2620
S4	Without oversampling	Boosting with RF	69.5187
	With oversampling	Bagging with J48 using Laplace	74.3316

**Table 8:** Summary of the best results for the prediction of mortality (Precision, F-measure, Recall).

Scenario	Data Aproach	Classifier	Precision	F-Measure	Recall
S1	Without oversampling	Bagging with RF	0.702	0.705	0.718
	With oversampling	Boosting with J48 using Laplace	0.738	0.731	0.738
S2	Without oversampling	SL	0.682	0.682	0.685
	With oversampling	J48	0.744	0.743	0.743
S3	Without oversampling	Bagging with SL	0.683	0.688	0.698
	With oversampling	Bagging with SL	0.727	0.726	0.727
S4	Without oversampling	AdaBoost + RF	0.688	0.691	0.695
	With oversampling	Bagging with J48 using Laplace	0.742	0.742	0.743

**Table 9:** Summary of the best AUC results for the prediction of mortality.

Scenario	Data Approach	Classifier	AUC
S1	Without oversampling	AdaBoost with RF	0.821
	With oversampling	AdaBoost with RF	0.862
S2	Without oversampling	BN	0.820
	With oversampling	Bagging with RF	0.879
S3	Without oversampling	BN	0.820
	With oversampling	RF	0.805
S4	Without oversampling	BN	0.812
	With oversampling	Bagging with RF	0.864

**Table 10:** Best results obtained in this study.

Data Mining Models	Scenario	Data Approach	Classifier	Accuracy (%)	Precision	F-Measure	Recall
DMM1	S2	With oversampling	J48	74.3316	0.744	0.743	0.743
DMM2	S1	With oversampling	RF	83.2599	0.833	0.833	0.833
